# Genetic Variability as a Regulator of TLR4 and NOD Signaling in Response to Bacterial Driven DNA Damage Response (DDR) and Inflammation: Focus on the Gastrointestinal (GI) Tract

**DOI:** 10.3389/fgene.2017.00065

**Published:** 2017-05-29

**Authors:** Evagelia Spanou, Polyxeni Kalisperati, Ioannis S. Pateras, Alexandros Papalampros, Alexandra Barbouti, Athanasios G. Tzioufas, Athanassios Kotsinas, Stavros Sougioultzis

**Affiliations:** ^1^Gastroenterology Division, Department of Pathophysiology, “Laikon” General Hospital, University of AthensAthens, Greece; ^2^Department of Histology and Embryology, University of AthensAthens, Greece; ^3^1st Department of Surgery, “Laikon” General Hospital, University of AthensAthens, Greece; ^4^Department of Anatomy-Histology-Embryology, University of IoanninaIoannina, Greece; ^5^Department of Pathophysiology, “Laikon” General Hospital, University of AthensAthens, Greece

**Keywords:** toll-like receptors (TLRs), nod-like receptors (NLRs), DNA damage response (DDR), single nucleotide polymorphism (SNP), mutation, inflammation and tumorigenesis

## Abstract

The fundamental role of human Toll-like receptors (TLRs) and NOD-like receptors (NLRs), the two most studied pathogen recognition receptors (PRRs), is the protection against pathogens and excessive tissue injury. Recent evidence supports the association between TLR/NLR gene mutations and susceptibility to inflammatory, autoimmune, and malignant diseases. PRRs also interfere with several cellular processes, such as cell growth, apoptosis, cell proliferation, differentiation, autophagy, angiogenesis, cell motility and migration, and DNA repair mechanisms. We briefly review the impact of TLR4 and NOD1/NOD2 and their genetic variability in the process of inflammation, tumorigenesis and DNA repair, focusing in the gastrointestinal tract. We also review the available data on new therapeutic strategies utilizing TLR/NLR agonists and antagonists for cancer, allergic diseases, viral infections and vaccine development against both infectious diseases and cancer.

## Introduction – Innate Immune System and Genomic Variability

**FIGURE 1:**
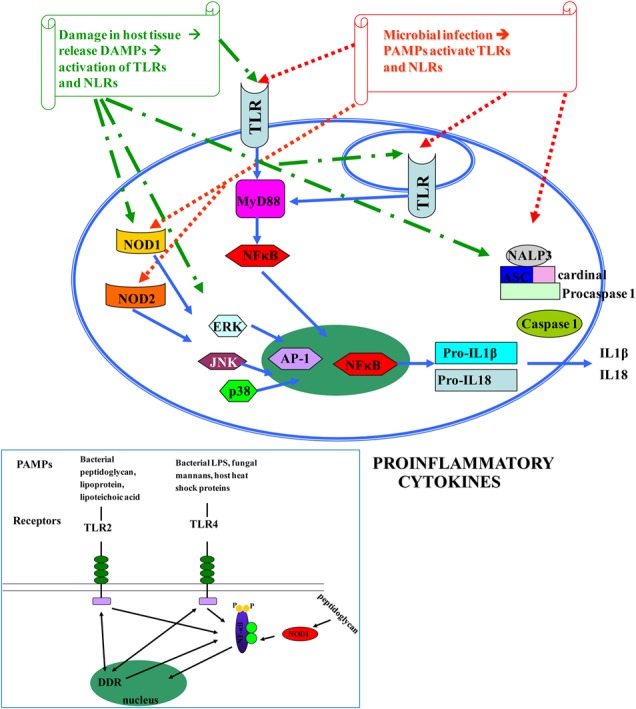
**Pathogen-associated molecular patterns (PAMPs) [microbial nucleic acids, including DNA (e.g., unmethylated CpG motifs), double-stranded RNA (dsRNA), single-stranded RNA (ssRNA), 5′-triphosphate RNA, lipoproteins, surface glycoproteins, and membrane components such as peptidoglycans, lipoteichoic acid, lipopolysaccharide (LPS), and glycosylphosphatidylinositol] and DAMPs [endogenous molecules normally found in cells and released during cell death, such as ATP, uric acid, the cytokine IL1a, heparin sulfate, RNA, and DNA] (Kawai and Akira, 2010; Tang et al., 2012) bind to TLRs and NLRs, activate NF-κB and AP-1 transcription factors and lead to the production of pro-inflammatory cytokines that perpetuate inflammation and induce tissue damage.** DAMPs are localized within the nucleus and cytoplasm (HMGB1), cytoplasm alone (S100 proteins), exosomes (heat shock proteins, HSP), the extracellular matrix (hyaluronic acid), and in plasma components such as complement (C3a, C4a, and C5a), but also, they can be mimicked by release of intracellular mitochondria, consisting of formyl peptides and mitochondrial DNA (with CpG DNA repeats) (Tang et al., 2012). Different TLRs serve as receptors for diverse ligands (Mitchell et al., 2007).

The human innate immune system is activated when pathogen recognition receptors (PRRs) recognize either pathogen-associated molecular patterns (PAMPs), or danger-associated molecular patterns (DAMPs) (Akira et al., 2006; Kawai and Akira, 2011). PRRs are in both cell membranes and cytosol of macrophages, fibroblasts, mast cells, dendritic cells, and circulating leucocytes (Newton and Dixit, 2012). They include members of the Toll-like receptors (TLRs), nucleotide-binding, and oligomerization domain containing receptors (NOD-like receptors, NLRs), retinoic acid-inducible gene(RIG) I-like RNA helicases, C-type lectins, and AIM2-like receptors(ALRs) (Saxena and Yeretssian, 2014) (**Figure 1**).

Available data have shown that genetic variability influences the susceptibility and evolution of several human diseases, like autoimmune diseases or infections, by affecting numerous cellular processes hence modulating the response to environmental and intrinsic factors (Orr and Chanock, 2008). Diseases associated with deficiencies in a single gene are not common in the population, therefore many epidemiological studies are now focused on the diversity of the contributing factors of complex illnesses (Orr and Chanock, 2008), responsible for most of the human morbidity and mortality. It is generally accepted that multiple genetic defects contribute to the phenotype of complex diseases, while the effects of single polymorphisms are usually veiled. Powerful tools such as high throughput expression profile analysis and genome-wide association studies (GWAS) are currently implemented to investigate the different polymorphisms and their interactions that culminate to disease development (Mayerle et al., 2013; Kim et al., 2014).

In this review, we highlight the impact of genetic diversity encoded in the *TLR4* and *NOD1/NOD2* loci to the progression of inflammation, tumorigenesis and the process of DNA repair, focusing in the gastrointestinal tract.

## Toll-Like Receptors

There are 10 members of TLRs, type I transmembrane glycoproteins, in humans (TLR1–TLR10) (Janssens and Beyaert, 2003). Their extracellular domain contains leucine-rich repeats (LRRs) expressed by cells of the innate immune system, which are involved in ligand binding (Bowie and O’Neill, 2000), while the intracellular tail contains a Toll/interleukin (IL)-1 receptor (TIR), that mediates downstream signaling. TLRs are well conserved across species and were first described in *Drosophila* (Medzhitov et al., 1997). They recognize bacterial and viral PAMPs in the extracellular environment (TLR1, TLR2, TLR4, TLR5, TLR6) or endolysosomes (TLR3, TLR7, TLR8, TLR9, TLR10) (O’Neill, 2006). Different TLRs serve as receptors for diverse ligands (Mitchell et al., 2007). TLRs are essential for the initiation of protective immunity against infections. Nevertheless, aberrant TLR responses may contribute to inappropriate acute and chronic inflammation and to systematic autoimmune diseases. In addition, it has become apparent that endogenous molecules released by dying cells or by some pathological conditions activate TLRs, further promoting inflammatory or autoimmune diseases (Kawai and Akira, 2010; Koberlin et al., 2016) (**Figure 1**). Despite the extensive study of TLRs in the gastrointestinal tract, the exact location and function of individual TLRs in various disease states is still evolving (Fukata and Abreu, 2008).

TLR4 is an essential member of the TLR family, which responds to bacterial lipopolysaccharide(LPS), a component of the outer membrane of Gram(–) bacteria(Akira et al., 2006).

## TLR4 Polymorphisms

Recent studies, conducted in several populations, have shown associations between *TLR* polymorphisms and the risk of gastric cancer (GC) (**Table 1A**). Some of these polymorphisms, such as *TLR4*rs4986790 (Asp299Gly), *TLR4*rs4986791 (Thr399Ile), *TLR4* rs10759932, *CD14* -260C/T, and *TLR2*-196to-174del appear to be associated with gastric precancerous lesions which may lead to intestinal type GC (Castano-Rodriguez et al., 2013). Especially two of the above polymorphisms, *TLR4*rs4986790 and *TLR4*rs4986791, disrupt the normal structure of the extracellular domain of TLR4, resulting in a protein with reduced binding affinity to the ligands of *Helicobacter pylori* (El-Omar et al., 2008).

**Table 1A:** Genetic polymorphisms in the *TRL4* signaling pathway that have been studied in relation to gastric cancer and CRC.

Polymorphism	Reference	Type of cancer	Sample size/population	OR/ 95% CI
rs4986790 (Asp299Gly)	Qadri et al., 2013	GC	330/ Indian	1,15 /0,66–2,03
rs4986790 (Asp299Gly)	de Oliviera et al., 2013	GC	440/ Brazilian	2,01 /1,06–3,81
rs4986790 (Asp299Gly)	Santini et al., 2008	GC	322/ Italian	0,97 /0,37–1,14
rs4986790 (Asp299Gly)	Hold et al., 2007	GC	395/ Caucasian	2,10/1,10–4,20
rs4986791(Thr399Ile)	Qadri et al., 2013	GC	330/ Indian	1,39/0,70–2,78
rs4986791(Thr399Ile)	de Oliviera et al., 2013	GC	440/ Brazilian	1,81 /0,64–5,15
rs4986791(Thr399Ile)	Santini et al., 2008	GC	322/ Italian	3,62 /1,27–6,01
rs10116253	Castano-Rodriguez et al., 2013	GC	310/ Chinese	0,56 /0,34–1,00
rs10759931	Castano-Rodriguez et al., 2013	GC	310/ Chinese	0,56 /0,33–0,97
rs10759932	Castano-Rodriguez et al., 2013	GC	310/ Chinese	0,59 /0,34–1,04
rs10983755	Kim et al., 2013	GC	974/ Korean	1,41 /1,01–1,97
rs11536889	Kupcinskas et al., 2011	GC	349/ Caucasian	1,03 /0,62–1,71
rs1927911	Huang et al., 2014	GC	511/ Chinese	0,37 /0,21–0,70
rs2149356	Castano-Rodriguez et al., 2013 (170)	GC	310/ Chinese	0,59 /0,34–1,02
rs10759931 GG vs AA+GA	Sheng et al., 2015	CRC	1198 cases + 1290 controls Asian and caucasian	1,95/1,00–3,77
Thr399Ile TT vs CC	Sheng et al., 2015	CRC	619 cases + 632 controls Asian and caucasian	4,99/1,41–17,65
Thr399Ile C carriers	Sheng et al., 2015	CRC	619 cases + 632 controls Asian and caucasian	4,50/1,27–15,87
rs10759931	Semlali et al., 2016	CRC	115 case + 112 controls/Saudi Arabian	0.086/0.04–0.18
rs10759931	Pimentel-Nunes et al., 2013	CRC	193 cases + 278 controls/Portugueses	3.30/1.18–9.28

*GS, Gastric cancer; CRC, Colorectal cancer; OR, Odds ratio; NS, Not specified; CI, confidence intervals.*

**Table 1B:** Genetic polymorphisms in the NOD-like receptor signaling pathway that have been studied in relation to gastric cancer.

Polymorphism	Reference	Type of cancer	Sample size/population	OR/ 95% CI
NLRP3 rs3806265	Castaño-Rodríguez et al., 2014a,b	GC	310/Chinese	3.33/1.09–10.13
NLRX1 rs10790286	Castaño-Rodríguez et al., 2014a,b	GC	310/Chinese	4,00/1,66–9,61
NOD2 rs7202124	Companioni et al., 2014	GC	1649/Caucasian	0,97/0,37–1,14
NOD1 rs2907749	Wang et al., 2012	GC	456/ Chinese	0,50/0,26–0,95
NOD1 rs5743336	Kupcinskas et al., 2011	GC	324/ Caucasian	1,01/0,48–2,16
NOD2 rs2066844 (R702W)	Angeletti et al., 2009	GC	326/Caucasian	4,1/1,75–9,42
NOD1 rs2075820 (E266K)	Hofner et al.,	GC	211/ Caucasian	1,06/0,66–1,73

*GC, gastric cancer; OR, odds ratio; CI, confidence intervals.*

Data are few regarding *TLR4* polymorphisms and *H. pylori*– associated diseases. Analyzing a population from Northern India, Achyut et al. (2007) concluded that *TLR4*rs4986791 substitution may be a risk factor for gastritis and precancerous lesions, while they reported a significant association between *TLR4*rs4986790 and neutrophil infiltration. In another study from Hungary, Hofner et al. (2007) found no association of TLR4 polymorphisms between *H. pylori* positive patients with or without gastritis or duodenal ulcers. Two studies in children by Tseng et al. (2006) and Moura et al. (2008), reported no association between *TLR4*rs4986790 and risk of infection. Based on the current evidence, it seems likely that these polymorphisms have a marginal or no impact in *H. pylori* acquisition risk and associated inflammation. However, a blunt IgA antibody production against *H. pylori* infection was observed in Greek patients with *TLR4* polymorphisms, suggesting that a defect or dysregulation of humoral mucosal defense may be present (Manolakis et al., 2010).

The lack of significant effects of the *TLR4* polymorphisms in infections is not uncommon among Europeans. Indo-European populations are frequently (6–14%) double heterozygous for both polymorphisms (Ferwerda et al., 2007), and *TLR4*rs4986790/*TLR4*rs4986791 haplotype may not functionally differ from wild type TLR4. Conversely, *TLR4*rs4986790 was frequently found (10–18%) among African populations, with only 2% having *TLR4*rs4986791 co-segregation (Bochud et al., 2009; Bhuvanendran et al., 2011). Disparities between Europeans (co-segregation) compared to Asian and African populations (lack of co-segregation) may explain the significant associations noted for endemic diseases in Asia and Africa.

Sato et al. (2012) reported that the genetic variation *TLR4*rs11536889 (+3725G/C) may contribute to the translational regulation of TLR4 and influences the response to LPS. According to Liu et al. (2011), *TLR4*rs10759932 decreases the expression of FOXP3, a marker for regulatory T (Treg) cells that are increased in *H. pylori* gastritis and probably contribute to *H. pylori* persistence (Jang, 2010).

Regarding colorectal cancer (CRC), Abuli et al. (2013) reported 20 susceptible SNPs in 18 risk loci for CRC, among which were TLR gene polymorphisms. The GG genotype of *TLR4*rs4986790 and the TT genotype of *TLR4*rs4986791 polymorphisms might be correlated with an increased risk of CRC, and may serve as biomarkers (Pimentel-Nunes et al., 2013; Sheng et al., 2015; Semlali et al., 2016) (**Table 1A**). In addition, a study by Wang et al. (2010) suggested that high immunohistochemical expression of TLR4 in colorectal tumors is associated with liver metastases and poor prognosis. In contrast, Nihon-Yanagi et al. (2012), support that TLR2 is mainly involved in colon tumorigenesis. Similar, apparently controversial results have been reported for other factors involved in gastrointestinal carcinogenesis (Evangelou et al., 2014). Taken together, we assume that TLRs are involved in colon cancer development and further work is needed to clarify their exact role.

## Nod-Like Receptors (NLRs)

The NLR family includes NODs, NLRPs (also called NALPs), IL-1β-converting enzyme (ICE)-protease activating factor (IPAF), neuronal apoptosis inhibitor factors (NAIPs), and MHC class II transactivator (CIITA)(Ting et al., 2006). These molecules are in the cytoplasm and survey for the presence of intracellular pathogens. In humans, there are 22 known NLRs associated with many diseases (Zhong et al., 2013; Kim et al., 2015). There are four distinct domains in every NLR: a central NACHT (NAIP, CIITA, HET-E, and TP-2), an N-terminal domain that facilitates oligomerization, the ligand sensing LRRs on the C-terminal and the effector domain, which may be pyrin domain (PYD), caspase recruitment domain family (CARD), or baculoviral IAP repeat (BIR). Each NLR contains different effector domain which mediates signal transduction to downstream targets leading to activation of inflammatory caspases by inflammasomes or NF-κB by NODs. NAIP contains BIR domain, IPAF, while some of the NALP family contain CARD domain and most NALPs contain PYD domain (Saxena and Yeretssian, 2014).

NOD1 (NLRC1) and NOD2 (NLRC2) were the first NLRs reported. NODs initiate the activation of MAPKs and NF-κB via interaction with serine-threonine kinase RICK and activation of kinase TAK1 (Inohara et al., 2005). These two molecules (NOD1-NOD2) are essential for tissue homeostasis and host defense against bacterial pathogens (Philpott et al., 2014). Interestingly, single-nucleotide polymorphisms (SNPs) in the NOD2 (CARD15) gene are considered as a significant risk factor in Crohn’s disease (Ogura et al., 2001). NOD1 is expressed in both hematopoietic and non-hematopoietic cells, while NOD2 is restricted to hematopoietic and some specialized epithelial cells, like Paneth cells of the small intestine (Ogura et al., 2003).

In addition, NODs seem to be essential for host defense against non-invasive Gram (-) bacteria, such as *H. pylori* (Viala et al., 2004). Upon activation, both NOD1 and NOD2 self-oligomerize and, through homotypic CARD-CARD interactions, recruit the CARD containing adaptor receptor-interacting protein kinase 2 (RIP2 or RIPK2), leading to the formation of a ‘Nodosome’, a multi-protein signaling complex that results in NF-κB and MAPK-mediated inflammatory and antimicrobial response (Magalhaes et al., 2011; Keestra et al., 2013). In addition, NLR activation leads to formation of a molecular scaffold complex termed inflammasome. Three human inflammasomes have been described based on the involved NLR protein: the NLP1, the NLP3 and the IPAF. All of them activate caspase-1, a protein essential for the transformation of the pro-IL-1β and pro-IL-18 to the mature cytokines IL-1β and IL-18, which play central role in inflammatory processes (Fukata et al., 2009) (**Figure 1**).

## NLR Polymorphisms

The four most studied polymorphisms of *NOD2* are: rs2066842C/T, rs2066844C/T, rs2066845C/G, rs2066847insC (**Table 1B**). As they are in coding region, they affect the function of NOD2, by altering the primary amino acid sequence (Liu et al., 2014). These four polymorphisms were initially associated with increased risk of Crohn’s disease (Hugot et al., 2001) and ulcerative colitis (Gazouli et al., 2005). Kurzawski et al. (2004) first linked *NOD2* polymorphisms with CRC. Subsequent studies were inconsistent regarding the association of the *NOD2* polymorphisms with risk of multiple cancers such as gastric, endometrial, breast, ovarian and laryngeal. A meta-analysis by Liu et al. (2014) suggested that *NOD2*rs2066844C/T, rs2066845C/G, and rs2066847insC polymorphisms may be associated with increased cancer risk, especially gastrointestinal (**Table 1B**). *NOD2* polymorphisms have been correlated with dysplastic changes of gastric mucosa in the presence of *H. pylori* (Hnatyszyn et al., 2010); carriers also have increased prevalence of early onset breast and lung cancer (Lener et al., 2006).

On the other hand, no mutations in the *NOD1* gene have been associated with intestinal inflammation or CRC. Oppositely, a study by Chen et al. (2008) in a murine model of colitis-associated colon cancer revealed a basic anti-tumorigenic function of intact NOD1. Nevertheless, *NOD1* polymorphisms have been associated with the development of atopic eczema, asthma and increased serum IgE concentration (Hysi et al., 2005), while polymorphisms in the intronic region of NOD1 have been linked with the age of IBD onset (McGovern et al., 2005).

## The DNA Damage Response (DDR)

It is vital for every cell to protect the integrity of all the encoded information it hosts and enable the accurate transfer of genetic material during cell division. Given that all human cells are exposed to a multitude of genotoxic insults, endogenous and exogenous (Jackson and Bartek, 2009), a highly conserved and advanced DNA recognition and repair network, against a variety of DNA lesions is in operation. The DDR is a complex network of molecular mechanisms, which identifies the genetic damage and induces biochemical pathways which cause cell cycle arrest (so-called control points, checkpoints), promotes repair of lesions in the genetic material, or, alternatively, proceeds to the activation of anti-tumor barriers, apoptosis and senescence (Halazonetis et al., 2008; Gorgoulis and Halazonetis, 2010; Evangelou et al., 2013; Velimezi et al., 2013).

Among all types of genetic damage, the double-stranded breaks (DSBs) constitute the greatest threat to the cell. The presence of DSBs results in the DDR activation having as a key effector the tumour-suppressor protein p53 (Rodier et al., 2007). DSBs can be induced by various stimuli such as ionizing radiation, activated oncogenes, or defective telomeres and are very harmful, even fatal, for the cell. Early activation of DDR in human precancerous lesions highlights the importance of this network in preventing cancer progression (Gorgoulis et al., 2005; Bartkova et al., 2006; Halazonetis et al., 2008; Gorgoulis and Halazonetis, 2010). However, continuous activation of DDR constitutes a sustained “pressure” eventually leading to the mutation of the *TP53* gene and loss of the anti-tumor barriers elicited by DDR, providing an explanation for the extremely high rate of *TP53* mutations in sporadic solid tumors and initiation of DDR in advanced cancers (Halazonetis et al., 2008; Negrini et al., 2010).

## The Interaction Between DDR and Immune System

Pathogen recognition receptors are major sensors of innate immunity but they also affect adaptive immune responses. In addition, many PRRs seem to interfere with several cellular processes such as cell growth, apoptosis, cell proliferation, differentiation, autophagy, angiogenesis, cell motility and migration (Kutikhin et al., 2014). Currently there is a strong interest in investigating the impact of PRRs in the process of DNA repair. Undoubtedly, DDR and the immune system are parts of the same protective mechanism aiming to maintain cellular integrity against endogenous and exogenous threats. DAMPs or PAMPs engagement to TLRs leads to DDR activation, by induction of activator protein-1(AP-1) and inflammatory mediators such as IL-12, IL-18, and IL-23, known downstream effectors of TLR signaling (Harberts and Gaspari, 2013). Nevertheless, aberrant activation of the protective mechanism can be harmful not only for the cellular, but also for the whole organismal systemic homeostasis resulting in chronic, even fatal, diseases. Indeed, the state of chronic inflammation observed in many pathologies, such as neoplasia and autoimmunity, may be partially attributed to persistent DDR stimulation (Pateras et al., 2015). From all the above it is conceivable that a common initiating point is potentially shared between malignancies, connective tissue diseases and infectious diseases.

The role of DDR in the pathogenesis of autoimmune diseases is well established (Solier and Pommier, 2014; Gunther et al., 2015; Souliotis et al., 2016). According to a recent report (Funabiki et al., 2014), lupus-like features were developed spontaneously in a mutant mouse line bearing MDA5 (RIG-I-like receptor) gain of function mutation in the absence of the appropriate viral ligand, thus providing direct evidence connecting dysregulation of PRRs with autoimmunity. Furthermore, it is well-established that chronic infection or chronic inflammation is a major driving force in 20% of human cancer and TLR/NLR signaling pathways serve as a link between chronic inflammation and cancer such as colorectal and other tumors (Wang et al., 2006; El-Omar et al., 2008; Goto et al., 2008; Lowe et al., 2010; Yang et al., 2010; Cui et al., 2014).

Based on recent reports (Wang et al., 2013; Ahmad et al., 2014), TLR4 may both upregulate and downregulate specific DNA repair proteins, in various ways in a cell specific manner. Intracellular TLRs, such as TLR7, TLR8, TLR9 stimulated by imidazoquinolines, ssRNA, anti-phospholipid antibodies, bacteria, viral CpG-DNA, and IgG-chromatin complexes (Kutikhin, 2011), signal via the protein encoded by myeloid differentiation primary response gene 88 (*MyD88*) and also modulate DNA repair in a specific manner. Regarding the NLRs, Licandro et al. (2013) reported that the ectodomain of NLRP3 recognizes certain DAMPs, leading to inflammasome formation and to the development of aseptic inflammation. Taken together, the above presented data imply that PRRs, and especially TLR4, TLR7, TLR8, TLR9, and NLRP3, may be important regulators of DNA repair machinery.

On the other hand, it is worth mentioning that DDR in turn, controls human TLR gene expression. Menendez et al. (2011) studied p53 responsiveness in primary human lymphocytes from healthy volunteers and found that most of the TLR genes respond to p53 via canonical as well as non-canonical promoter binding sites. They also observed considerable inter-individual variability suggesting that DNA and p53 metabolic stresses can diversely modulate the innate immune system as well as downstream cytokines.

## Targeting TLRs and NLRs-Therapeutic Implications

Toll-like receptor (TLR) agonists are being developed for the treatment of cancer, allergic diseases, viral infections, but also as adjuvants for vaccines against infectious diseases and cancer (Romagne, 2007) with considerable success. For example, BCG (Bacillus Calmette-Guerin) and Imiquimod, used as treatment for bladder cancer and basal cell carcinoma, respectively, contain several TLR agonists that contribute to their antineoplastic efficacy (Uehori et al., 2003; Geisse et al., 2004; Dunne et al., 2011; Vacchelli et al., 2012).

Monophosphoryl lipid A (MPL), a TLR4 agonist purified from *Salmonella Minnesota* LPS is used as an adjuvant, to enhance adaptive immune responses, in human licensed vaccines against papillomavirus (HPV) and hepatitis B virus (HBV) infections (Maisonneuve et al., 2014). Moreover, promising ongoing research in this field investigates the potential of other TLR agonists, either alone or in combination, as adjuvants in vaccines against bacterial, viral and neoplastic diseases (Cooper et al., 2008; Maisonneuve et al., 2014).

Agonists to TLR7/8/9, have been successfully tested in adults as novel therapeutics for allergies, asthma and allergic rhinitis, because they induce a strong Th1 response (Hennessy et al., 2010; Aryan et al., 2014). A single-stranded DNA-based synthetic oligodeoxynucleotide that activates TLR-9 in intestinal immunocytes, and induces the production of anti-inflammatory cytokines has been administered topically during lower GI endoscopy in patients with ulcerative colitis, refractory to standard therapy, with promising results (Atreya et al., 2016).

On the other hand, inappropriate TLR stimulation is observed in chronic idiopathic inflammatory and autoimmune diseases. Thus, TLRs antagonists aiming to attenuate the exaggerated inflammatory response have been tested for potential clinical benefit in acute and chronic infections, including sepsis, with variable success (Rossignol and Lynn, 2005; Opal et al., 2013; Savva and Roger, 2013).

TLR antagonists may also prove to be of benefit in treatment of autoimmune diseases, especially Systemic Lupus Erythematosus (SLE), although clinical data are not yet available (Kanzler et al., 2007; Wu et al., 2015). It is worth mentioning that hydroxychloroquine, an anti-malarial agent with acknowledged anti-inflammatory properties used for years as treatment of SLE and rheumatoid arthritis (RA), has been recently found that is a potent TLR inhibitor. TLR blockage has also been studied in acute respiratory distress syndrome (ARDS), acute lung injury, RA, asthma, myocardial ischemia reperfusion injury, inflammatory bowel diseases, and pain management (Dunne et al., 2011; Connolly and O’Neill, 2012).

In contrast to TLRs, the effect of NLR agonists or antagonists has not yet been tested in humans. Nevertheless, data from basic research show that manipulation of the NLR associated molecular pathways holds promise as future therapeutic target for the treatment of inflammation and cancer.

## Conclusion – Future Perspectives

Nonetheless, the pleiotropic actions, redundancy, complex interactions, and the possibility of functional mutations of the involved molecules should always be kept in mind when interpreting the outcome of any therapeutic attempt. Intuitively, augmenting a weak or attenuating an excessive inflammatory reaction, by targeted therapeutic interventions may fine-tune host’s response and control disease progression. As briefly outlined above, TLRs and NLRs are key molecules involved in the inflammatory process and suitable candidates for therapeutic manipulation. Available data thus far point out that their therapeutic potential has been only partially exploited.

Nonetheless, it must always be kept in mind the pleiotropic actions, redundancy, complex interactions and the possibility of functional mutations of the involved molecules, in order to interpret the outcome of any therapeutic attempt. Future research should shed more light on the complex evolving operation of the PRRs and the associated molecular pathways in various disease states, in order to timely select the appropriate targets for therapeutic intervention.

## Author Contributions

ES, PK, ISP, AP, AB, AGT, AK, SS designed the manuscript. ES, PK, ISP, AP, AB collected data, reviewed literature and generated tables and figures. AGT, AK and SS wrote and supervised the manuscript.

## Conflict of Interest Statement

The authors declare that the research was conducted in the absence of any commercial or financial relationships that could be construed as a potential conflict of interest.
